# Ten newly recorded species of xyleborine ambrosia beetles (Coleoptera, Curculionidae, Scolytinae, Xyleborini) from Thailand

**DOI:** 10.3897/zookeys.862.34766

**Published:** 2019-07-09

**Authors:** Wisut Sittichaya, Sarah M. Smith, Roger A. Beaver

**Affiliations:** 1 Department of Pest Management, Faculty of Natural Resources, Prince of Songkla University, Hat Yai, Songkhla, 90112, Thailand Prince of Songkla University Hat Yai Thailand; 2 Michigan State University, Department of Entomology, 288 Farm Lane, room 243, East Lansing, MI 48824, USA Michigan State University East Lansing United States of America; 3 161/2 Mu 5, Soi Wat Pranon, T. Donkaew, A. Maerim, Chiangmai, 50180, Thailand Unaffiliated Chiangmai Thailand

**Keywords:** Diversity, Oriental region, reinstated species, southern Thailand, xyleborines

## Abstract

Ten species of ambrosia beetles of the tribe Xyleborini, *Amasabeesoni* (Eggers, 1930), *Amasaopalescens* (Schedl, 1937), *Amasacylindrotomica* (Schedl, 1939), *Arixyleborushirsutulus* Schedl, 1969, *Beaveriumlatus* (Eggers, 1923), *Cnestusprotensus* (Eggers, 1930), *Coptodryasquadricostata* (Schedl, 1942), *Cryptoxyleborusconfusus* Browne, 1950, *Cryptoxyleboruspercuneolus* (Schedl, 1951) and *Cyclorhipidionvigilans* (Schedl, 1939), are recorded here for the first time in Thailand. Diagnostic characters, illustrations, distribution and biological data are provided for each species. *Xylosandrusramulorum* (Schedl, 1957), **stat. res.** is removed from synonymy with *Amasacylindrotomica* and reinstated as a valid species.

## Introduction

The Scolytinae is a subfamily of bark and wood-boring weevils which includes more than 6000 species (Alonso-Zarazaga and Lyal 2009), and is of considerable economic importance in both temperate and tropical regions. The majority of species attack dying or dead trees; a few, economically important species attack healthy or apparently healthy trees and can cause die-back or mortality. The Xyleborini is one of the largest tribes of Scolytinae with more than 1100 described species (Hulcr et al. 2015), and many more undescribed. All are inbreeding ambrosia beetles. They are wood-borers intimately associated with symbiotic ambrosia fungi upon which both adults and larvae feed in gallery systems constructed in the xylem (Beaver et al. 2014, Kirkendall et al. 2015). The female alone is responsible for gallery construction. The eggs are laid loose in the gallery, and the larvae develop freely in the maternal gallery feeding upon the ambrosia fungi growing on its walls. The sex ratio is strongly biased towards females, and sib-mating occurs within the maternal gallery prior to the emergence of the new generation via the original entrance hole (Kirkendall et al. 2015).

The first checklist of the Scolytinae of Thailand was that of Beaver and Browne (1975), which listed 33 species of Xyleborini, the majority collected in the north of the country. Further species of Xyleborini have been added by Beaver (1990, 1999, 2010), Beaver and Hulcr (2008), Sittichaya (2012), Sittichaya et al. (2012) and Beaver et al. (2014), bringing the current total number of xyleborine species recorded in Thailand to 146 (Beaver et al. 2014). The present paper records 10 further species of Xyleborini collected in southern Thailand, with diagnostic characters, collecting localities and information on host plants and biology where available. We expect many further species to be collected in this region. The scolytine fauna of the south of Thailand is continuous with the species-rich fauna of Malaysia and Indonesia, whilst that of Thailand north of the Isthmus of Kra on the Thai-Malay Peninsula at about 11‒13°N is more similar to that of the rest of South-East Asia (Cambodia, Laos, Myanmar, Vietnam) (Beaver et al. 2014).

## Material and methods

**Figure 1.**
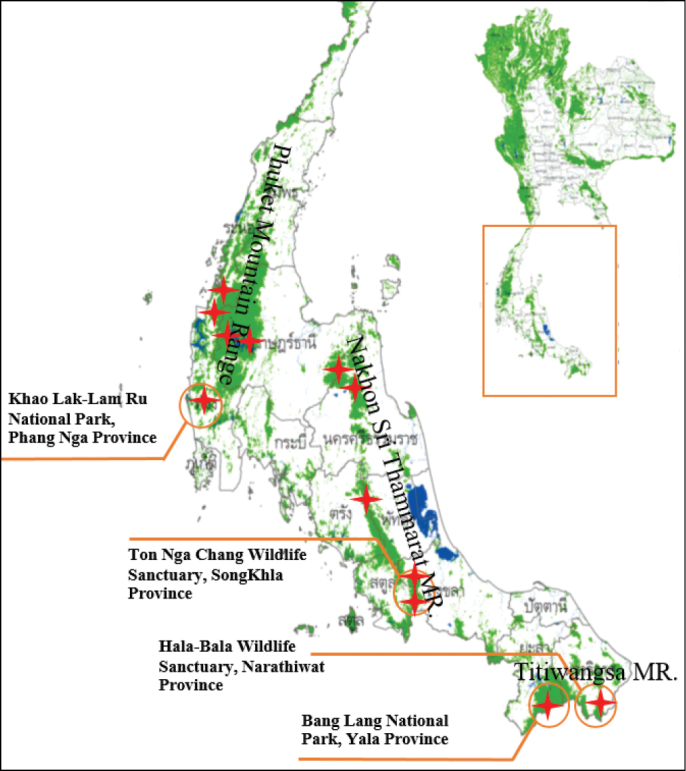
Peninsular Thailand showing forest covered areas (green), and conservation areas in which the beetles were trapped (stars). Labels indicate study areas where newly recorded species were captured. (Modified from http://new.forest.go.th/land/)

Specimens were collected from three forest complexes in peninsular southern Thailand over a thirteen to fifteen month trapping period. Ethanol-baited flight intercept traps were placed in 12 study sites in 10 conservation areas in the Titiwangsa Mountain Range, Nakhon Sri Thammarat Mountain Range and Phuket Mountain Range (Fig. 1). In the Titiwangsa Mountain Range, at the Hala-Bala Wildlife Sanctuary, 10 traps were deployed at one site from 1 May 2014 to 30 May 2015. In the Nakhon Sri Thammarat Mountain Range, 10 traps were placed at each of five sites from 1 October 2013 to 31 December 2014. In the Phuket Mountain Range, 10 traps were placed at each of five sites from 1 April 2014 to 30 April 2015. At each site, the traps were deployed in a transect line, 100 m apart, in mature forest 1 km from the surrounding agricultural areas or secondary forest. At the Bang Lang National Park in Yala Province, hand collecting from logs and branches was carried out for one week from 1‒7 February 2014. Trapped beetles were sorted and identified using a Leica stereomicroscope EZ4 and Leica S8 APO (Leica Microsystems Pte Ltd, Germany). Photographs were taken with a Canon 6D digital Camera with a Canon MP-E 65mm macro photo lens (Canon, Tokyo, Japan) and StackShot-Macrorail (Cognisys Inc, Michigan, USA). The photos were then combined with Helicon Focus 6.8.0. (Helicon Soft, Ukraine), and all photos were improved with Adobe Photoshop CS6 (Adobe Systems, California, USA). Plant names and classification follow http://www.theplantlist.org/. All specimens of the newly recorded species are currently deposited in the collection of W. Sittichaya at Prince of Songkla University. Duplicate specimens will subsequently be deposited in other museum collections.

## New records

### Genus *Amasa* Lea, 1893: 322

#### Amasa beesoni

Taxon classificationAnimaliaColeopteraCurculionidae

(Eggers, 1930)

Fig. 2A–F

Pseudoxyleborus beesoni Eggers 1930: 207.
Amasa beesoni (Eggers): Wood 1984: 223.

##### Diagnosis.

Large, 4.60−4.79 mm (*N* = 2) long; stout, 2.0−2.1 times longer than wide; body smooth, shining nearly glabrous, yellowish brown to dark brown in color; eye completely divided; antennal club with sutures obscured (type 5; Hulcr et al. 2007); pronotum from dorsal view rounded (type 1; Hulcr et al. 2007), anterior margin broadly round, anterior half finely asperate, 1.1 times wider than long; elytra 1.1 times longer than pronotum, elytral disc punctures very fine, confused, never seriate, lateral sides subparallel, widest on declivital summit, declivital summit at first interstriae bearing a pair of small flattened teeth, declivital face shining, striae impressed, interstriae finely, densely punctate.

**Figure 2.**
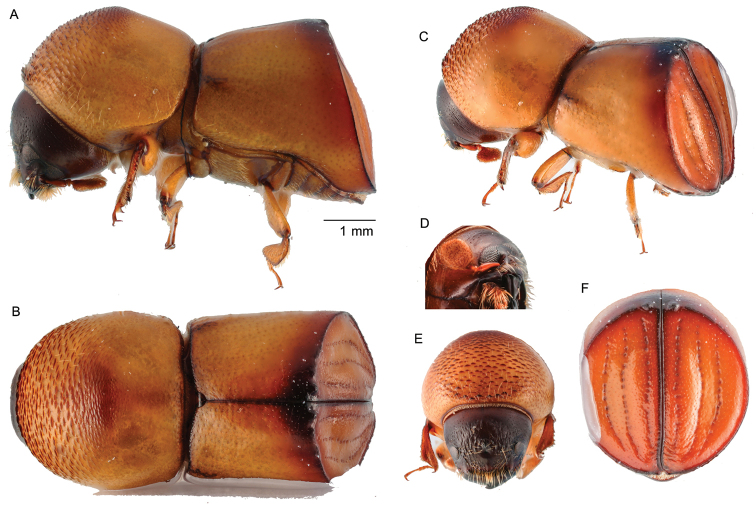
*Amasabeesoni* (Eggers, 1930) **A** lateral view **B** dorsal view **C** posterolateral view **D** antenna **E** front **F** declivity.

##### Material examined.

THAILAND, Khao Lak-Lam Ru National Park, Phang Nga Province, 8°39'22.4"N 98°17'31.6"E, tropical rainforest, ethanol-baited trap, 01.v.2015 (1), 01.iv.2015 (1), (W. Sittichaya).

##### Distribution.

Indonesia, Malaysia, Myanmar. New to Thailand.

##### Biology.

Recorded from *Dimocarpuslongan* Lour and *Xerospermumintermedium* Radlk. (Sapindaceae) and possibly with a fixed association with this family (Browne 1961). The gallery system, as in other *Amasa* species, comprises a short radial tunnel leading to a single, large, flat brood chamber, extending in the longitudinal plane (Browne 1961).

##### Remarks.

This species can be distinguished from all other *Amasa* recorded in Thailand by the completely divided eye and the small teeth at the apex of the elytral disc on the first interstriae.

#### Amasa cylindrotomica

Taxon classificationAnimaliaColeopteraCurculionidae

(Schedl, 1939)

Fig. 3A–E

Pseudoxyleborus cylindrotomicus Schedl, 1939: 40.
Xyleborus cylindrotomicus (Schedl): Schedl 1942: 6.
Xylosandrus cylindrotomicus (Schedl): Wood 1989: 177.
Amasa cylindrotomica (Schedl): Dole and Cognato 2010: 525.
Synonyms: Xyleborussemitruncatus Schedl, 1942: 35. Synonymy: Schedl 1951: 79; Wood 1989: 177.
Xyleborus truncatellus Schedl, 1951: 79. Synonymy: Kalshoven 1959: 95.
Xyleborus jucundus Schedl, 1954: 138 (new name for Xyleborustruncatellus Schedl, 1951 non Schedl 1949). Synonymy: Kalshoven 1959: 95.

##### Diagnosis.

Small, 2.1 mm (*N* = 1) long; stout, 2.0 times longer than wide; body shining, nearly glabrous, yellowish brown to brown in color; eye deeply emarginate; antennal club with sutures obscured (type 5; Hulcr et al. 2007); pronotum from dorsal view round (type 1; Hulcr et al. 2007) front broadly convex, from lateral view round near (type 1; Hulcr et al. 2007), anterior half of pronotum densely, finely asperate, base very finely punctate; elytra 1.07 times as long as pronotum, sides subparallel, widest at declivital summit, declivity dull, glabrous, strial punctures seriate, first stria straight, second and third laterally diverging, interstriae shagreened, two times broader than striae.

**Figure 3.**
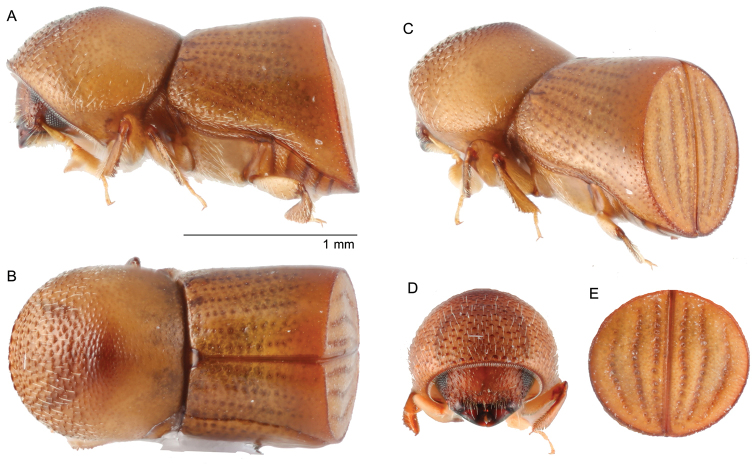
*Amasacylindrotomica* (Schedl, 1939) **A** lateral view **B** dorsal view **C** posterolateral view **D** front **E** declivity.

##### Material examined.

THAILAND, Ton Nga Chang Wildlife Sanctuary, Songkhla Province, 6°59'32.1"N 100°08'57.8"E, tropical rainforest, ethanol-baited trap, 01.ii.2014 (1) (W. Sittichaya).

##### Distribution.

Indonesia (Java, Sumatra). New to Thailand.

##### Biology.

Recorded only from *Syzygiumaromaticum* Merr & LM Perry (clove) (Myrtaceae).

##### Remarks.

This species can be distinguished from all other *Amasa* recorded in Thailand by its small size (2.1 mm long) and stout appearance; elytra approximately as long as the pronotum, and elytral declivity dull, glabrous.

It should be noted that the Afrotropical species, *Xyleborusramulorum* Schedl, 1957, included as a synonym of *A.cylindrotomica* by Wood (1989), Wood and Bright (1992), and Dole and Cognato (2010) is a different species. *Xyleborusramulorum* was described and figured from three specimens collected by Schedl in what was then Belgian Congo (Schedl 1957). Schedl (1963) gave some additional biological information, and figured the gallery system. Nunberg (1963) re-examined the holotype in the Royal Museum for Central Africa, Tervuren, and provided additional morphological characters. He also noted (Nunberg 1963) that the holotype was badly damaged, and that Schedl had evidently retained undamaged paratypes in his own collection. Browne (1965) transferred the species to *Xylosandrus* Reitter, 1913. Wood (1989) synonymised this Afrotropical species with the Oriental species, *Xylosandruscylindrotomicus* Schedl, 1939, without providing any reason for the synonymy. Dole and Cognato (2010) accepted the synonymy without examining type material, and listed the species as a synonym of *Amasacylindrotomica* (Schedl). Examination of an undamaged paratype (NHMW) clearly indicates that the species should be returned to *Xylosandrus* as a distinct species. *Xylosandrusramulorum***stat. res.** shares numerous characteristics with *Xylosandrus* including: mesonotal mycangial tuft present but unlike other *Xylosandus* the mycangium opening is on the pronotal disc rather than the pronotal base; truncate antennal club with segment 1 encircling the anterior face (type 1; Hulcr et al. 2007); pronotal anterior margin serrate; pronotum from lateral view with disc as long or longer than anterior slope (type 7; Hulcr et al. 2007); and elytral declivity truncate with 5 granulate striae on declivital face, interstriae also granulate. By comparison, *Amasa* species have the following characteristics (Hulcr and Smith 2010; Smith et al. in prep.): mesonotal mycangial tuft absent; flat antennal clubs with segment 1 never encircling the anterior face (types 3,4,5; Hulcr et al. 2007), pronotal anterior margin never serrate; pronotum from lateral view basic or robust (types 1 and 5; Hulcr et al. 2007); elytral declivity truncate with no more than 3 punctate striae on declivital face. Ventral characters, including the separation of the procoxae and protibia shape and sculpturing, were not described by Schedl and are not visible on the card mounted paratype. Based on the characteristics listed above, *Xyleborusramulorum* is here transferred to *Xylosandrus* where it shares features with the Asian species included in the *Xylosandruss.l.* clade (Dole and Cognato 2010): *X.beesoni* Saha, Maiti & Chakraborti, 1992, *X.borealis* Nobuchi, 1981, *X.brevis* (Eichhoff, 1877), *X.discolor* (Blandford, 1898), *X.diversipilosus* (Eggers, 1941), *X.jaintianus* (Schedl, 1967), *X.subsimilis* (Eggers, 1930) and *X.subsimiliformis* (Eggers, 1939).

#### Amasa opalescens

Taxon classificationAnimaliaColeopteraCurculionidae

(Schedl, 1937)

Fig. 4A–E

Xyleborus opalescens Schedl, 1937: 550
Amasa opalescens (Schedl): Wood and Bright 1992: 683.

##### Diagnosis.

Large, 4.5 mm long (*N* = 1); moderately stout, 2.5 times longer than wide; body shining, declivity opalescent, brown in color; eye deeply emarginated, almost completely divided; antennal club with first segment smaller than second (type 4; Hulcr et al. 2007); pronotum from dorsal view round (type 1; Hulcr et al. 2007), from lateral view tall (type 2; Hulcr et al. 2007), anterior margin round, armed with 6 small asperities, anterior portion of pronotum densely asperate, base shagreened; elytra 1.24 times longer than pronotum, lateral sides subparallel, widest at declivital summit, disc shining, striae minutely punctate, interstriae four times broader than striae, broadest at declivital summit, declivital margin unarmed, declivity smooth,opalescent, strial punctures large, irregularly spaced.

**Figure 4.**
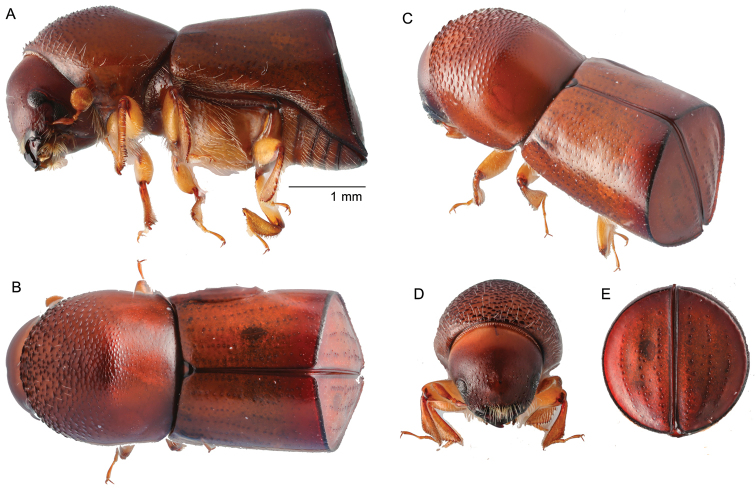
*Amasaopalescens* (Schedl, 1937) **A** lateral view **B** dorsal view **C** posterolateral view **D** front **F** declivity.

##### Material examined.

Thailand, Bang Lang National Park, Yala Province, Thailand-Malaysia border, 5°48'51.8"N 101°17'14.7"E, ex. small branches of unknown tree, 01.ii.2014 (1).

##### Distribution.

‘Borneo’, East and West Malaysia, Vietnam. New to Thailand.

##### Biology.

Recorded from *Eugenia* sp. and *Tristania* sp. (Myrtaceae), and possibly with a fixed association with this family (Browne 1961).

##### Remarks.

This species can be distinguished from all other *Amasa* recorded in Thailand by its large size (4.5 mm), moderately stout form (2.5 times longer than wide), declivital summit entirely carinate without teeth on first interstriae, declivity smooth, subshining, opalescent, strial punctures large, irregularly spaced, and eye deeply emarginated, almost completely divided.

### *Arixyleborus* Hopkins, 1915: 59.

#### Arixyleborus hirsutulus

Taxon classificationAnimaliaColeopteraCurculionidae

Schedl, 1969

Fig. 5A–E

Arixyleborus hirsutulus Schedl, 1969: 212.

##### Diagnosis.

Small, 2.0 mm (*N* = 1) long; 2.27 times longer than wide; pronotum shining, elytra densely setose, dark brown to black in color; pronotum from dorsal view with sides parallel, weakly elongate and rounded frontally (type 7; Hulcr et al. 2007), from lateral view elongate with low summit (type 7; Hulcr et al. 2007); elytra 1.25 times longer than pronotum, disc weakly convex, apical three-fourths rugose, striae and interstriae covered with small equally size granules, never forming strial furrows and interstrial ridges, elytra densely covered with setae, setae increasing in density towards apex, posterolateral carina oblique, granulate.

**Figure 5.**
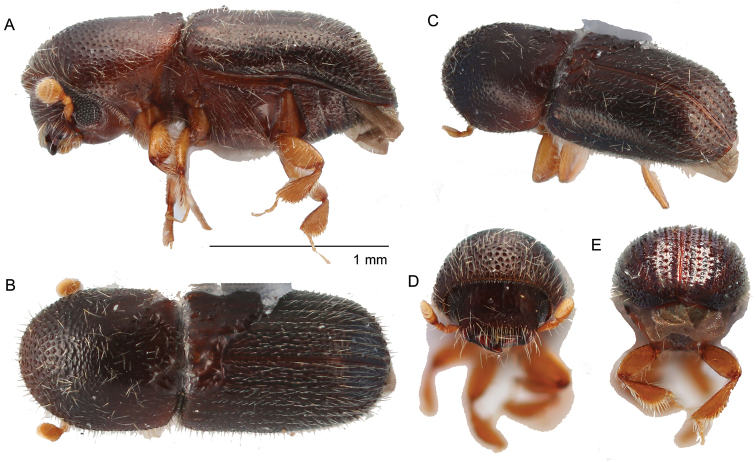
*Arixyleborushirsutulus* Schedl, 1969 **A** lateral view **B** dorsal view **C** posterolateral view **D** front **E** declivity.

##### Material examined.

THAILAND, Hala-Bala Wildlife Sanctuary, Narathiwat Province, lowland tropical rainforest, 5°47'44"N, 101°50'07"E, 01.iii.2015 (1), ethanol-baited trap (W. Sittichaya).

##### Distribution.

Philippines; imported to Japan from Borneo and Indonesia (Maluku). New to Thailand.

##### Hosts.

*Anisoptera* sp., *Dipterocarpus* sp., *Dryobalanops* sp., *Shorea* spp. (Dipterocarpaceae), *Artocarpus* sp. (Moraceae), and an unidentified species of Sapotaceae (Ohno 1990).

##### Remarks.

This species can be distinguished from all other *Arixyleborus* recorded in Thailand by the elytral striae and interstriae covered with small equally sized granules and without strial furrows and interstrial ridges, elytra densely setose with the setae increasing in density toward the apex.

### *Beaverium* Hulcr & Cognato, 2009: 25

#### Beaverium latus

Taxon classificationAnimaliaColeopteraCurculionidae

(Eggers, 1923)

Fig. 6A–F

Xyleborus latus Eggers, 1923: 177.
Terminalinus latus (Eggers): Wood 1986: 267.
Beaverium latus (Eggers): Hulcr and Cognato 2009: 26.

##### Diagnosis.

Large, 6.6 mm long (*N* = 1); stout, 2.2 times longer than wide; body covered with golden setae, setae longer on declivity, reddish brown to dark brown in color; pronotum from dorsal view conical (type 0; Hulcr et al. 2007), anterior half densely asperate, asperities robust, base asperate, anterior margin armed with two medium serrations, from lateral view appearing rounded and robust (type 5; Hulcr et al. 2007); elytra 1.35 times longer than pronotum, disc flat, weakly impressed, declivital posterolateral margins carinate, declivity flat, densely covered with long erect golden hair-like setae.

**Figure 6.**
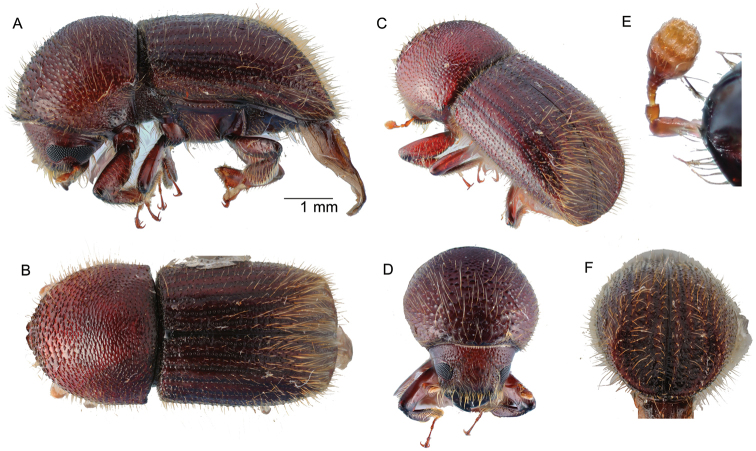
*Beaveriumlatus* (Eggers, 1923) **A** lateral view **B** dorsal view **C** posterolateral view **D** front **E** antenna **F** declivity.

##### Material examined.

THAILAND, Hala-Bala Wildlife Sanctuary, Narathiwat Province, lowland tropical rainforest, 5°47'44"N, 101°50'07"E, 01.v.2015 (1), ethanol-baited trap (W. Sittichaya).

##### Distribution.

‘Borneo’, East and West Malaysia, Indonesia (Sumatra). New to Thailand.

##### Biology.

Recorded from *Maranthescorymbosa* Blume (Chrysobalanaceae), *Shoreabalanocarpoides* Symington, *S.leprosula* Miq., S*horea* sp. (Dipterocarpaceae), *Intsiapalembanica* Miq. (Fabaceae), *Castanopsisinermis* (Lindl.) Benth. & Hook.f., *Lithocarpussundaicus* (Blume) Rehder (Fagaceae) (Browne 1961).

##### Remarks.

This species can be distinguished from all other *Beaverium* recorded in Thailand by the body brown to dark brown in color, declivital posterolateral margins carinate, declivity flat, and densely covered with long golden setae.

### *Cnestus* Sampson, 1911: 383

#### Cnestus protensus

Taxon classificationAnimaliaColeopteraCurculionidae

(Eggers, 1930)

Fig. 7A–F

Xyleborus protensus Eggers, 1930: 201.
Cnestus protensus (Eggers): Schedl 1958: 145.

##### Diagnosis.

Large, 4.0 mm long (*N* = 1); stout, 2.0 times longer than wide; body strongly shining, glabrous, black in color; pronotum from dorsal view conical frontally (type 6; Hulcr et al. 2007), pronotal apex strongly produced, armed with numerous strong serrations, anterior portion of pronotum strongly asperate, lateral margins parallel from the base to the middle, base densely coarsely punctate; mesonotal mycangial tuft absent on pronotal base; elytra round, elytral declivity strongly rounded and convex.

**Figure 7.**
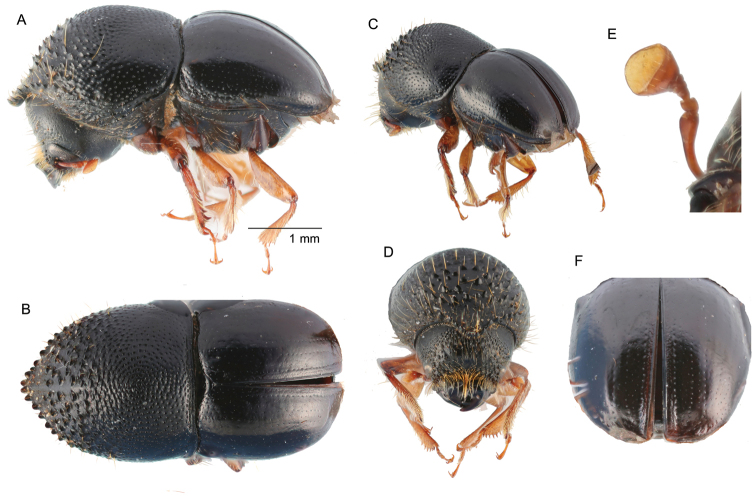
*Cnestusprotensus* (Eggers, 1930) **A** lateral view **B** dorsal view **C** posterolateral view **D** front **E** antenna **F** declivity.

##### Material examined.

THAILAND, Khao Lak-Lam Ru National Park, Phang Nga Province, 8°39'22.4"N, 98°17'31.6"E, tropical rainforest, ethanol-baited trap, 01.xii.2014 (1) (W. Sittichaya).

##### Distribution.

India (Meghalaya), Indonesia (Java). New to Thailand.

##### Biology.

Unknown. *Cnestus* species, as far as is known, are twig and shoot-borers, and the gallery system is typical of such species with a short radial or circumferential gallery running to the middle of the stem, and longitudinal branches up and down the stem in which the brood develop (Browne 1961, Hulcr and Cognato 2013).

##### Remarks.

This species can be distinguished from all other *Cnestus* recorded in Thailand by the strongly produced pronotal apex armed with strong serrations, pronotal base without a mycangial tuft, elytral declivity strongly rounded and convex. This species most closely resembles *C.nitidipennis* (Schedl), and can be distinguished by the distinctly larger size, much larger, coarser and more numerous pronotal apical serrations, punctures on pronotal base clearly coarser and denser, and sides of pronotum parallel for approximately half of the total length.

### *Coptodryas* Hopkins, 1915: 54

#### Coptodryas quadricostata

Taxon classificationAnimaliaColeopteraCurculionidae

(Schedl, 1942)

Fig. 8A–E

Xyleborus quadricostatus Schedl, 1942: 30.
Coptodryas quadricostata (Schedl): Wood and Bright, 1992: 826.

##### Diagnosis.

Moderately sized, 3.0 mm (*N* = 1) long; stout 2.0 times longer than wide; body moderately setose, brown to dark brown in color; pronotum from dorsal view round and robust (type 5; Hulcr et al. 2007), from lateral view round (type 1; Hulcr et al. 2007), anterior margin with a row of serrations, anterior half asperate, base shagreened, mesonotal mycangial tuft present along the base; elytra 1.53 times longer than pronotum, base covered with elytral mycangial tuft of setae, disc shining, covered with long golden setae, striae and interstriae 1,3,5 deeply depressed, interstriae 2,4 weakly elevated from middle of elytral and narrower behind forming horizontal sharp spines and extending beyond declivital summit, declivity densely covered with long soft hair-like strial setae and shorter interstrial setae.

**Figure 8.**
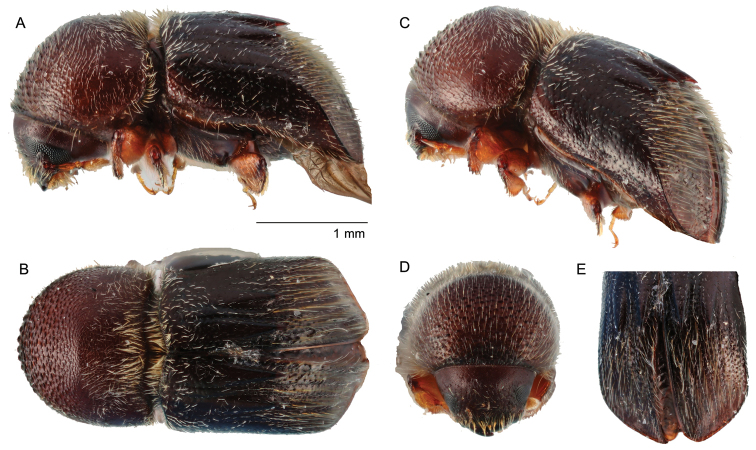
*Coptodryasquadricostata* (Schedl, 1942) **A** lateral view **B** dorsal view **C** posterolateral view **D** front **E** declivity.

##### Material examined.

THAILAND, Hala-Bala Wildlife Sanctuary, Narathiwat Province, lowland tropical rainforest, 5°47'44"N, 101°50'07"E, 01.i.2015 (1), ethanol-baited trap (W. Sittichaya).

##### Distribution.

‘Borneo’, East and West Malaysia, Indonesia (Java). New to Thailand.

##### Biology.

Recorded from *Campnosperma* sp. (Anacardiaceae), *Shorealeprosula* Miq., *S.parvifolia* Dyer (Dipterocarpaceae), *Elaeocarpus* sp. (Elaeocarpaceae), and *Garcinia* sp. (Clusiaceae). Browne (1961) notes that the species attacks small branches (1‒5 cm diameter). The gallery system usually encircles the stem, and has 1‒2 longitudinal branches in which the larvae develop (Browne 1961).

##### Remarks.

This species can be distinguished from all other *Coptodryas* recorded in Thailand by the declivital summit with four sharp spines extending beyond the summit.

### *Cryptoxyleborus* Wood & Bright, 1992: 828

#### Cryptoxyleborus confusus

Taxon classificationAnimaliaColeopteraCurculionidae

Browne, 1950

Fig. 9A–D

Cryptoxyleborus confusus Browne, 1950: 644.

##### Diagnosis.

Small, 2.0 mm long (*N* = 1); very elongate, 3.3 times longer than wide; body nearly glabrous, light brown in color; pronotum elongated basic shape (type 7; Hulcr et al. 2007), anterior margin rounded, convex, armed with a row of five serrations, anterior half of pronotum asperate, asperities tiny, pronotal base shagreened; elytra 1.71 times longer than pronotum, 1.87 times longer than wide, elytra abruptly tapering at apical third, interstriae reticulate-punctate, punctures shallow, very dense at the base, area of dense punctures broader at suture, discal punctures confused.

**Figure 9.**
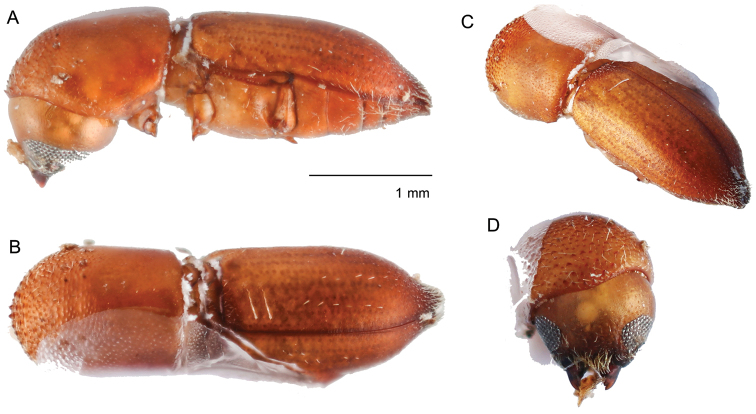
*Cryptoxyleborusconfusus* Browne, 1950 **A** lateral view **B** dorsal view **C** posterolateral view **D** front.

##### Material examined.

THAILAND, Ton Nga Chang Wildlife Sanctuary, Songkhla Province, 6°59'32.1"N 100°08'57.8"E, tropical rainforest, ethanol-baited trap, 01.iv.2014 (1) (W. Sittichaya).

##### Distribution.

Brunei Darussalam, East and West Malaysia, Indonesia (Sumatra). New to Thailand.

##### Biology.

Recorded from several species of *Shorea* (Dipterocarpaceae) (Browne 1961, Beaver and Hulcr 2008). Browne (1961) notes that the gallery system differs from the usual pattern found in the genus. A surface brood chamber is constructed between bark and wood in which most of the larvae develop. However, there are also more deeply penetrating tunnels into the wood.

##### Remarks.

This species is closely related to *C.vestigator* Schedl, which has the elytra more strongly posteriorly tapered and is more strongly shining in appearance. *Cryptoxyleborusconfusus* seems to be somewhat morphologically variable and DNA could show that the species is not monophyletic.

#### Cryptoxyleborus percuneolus

Taxon classificationAnimaliaColeopteraCurculionidae

(Schedl, 1951)

Fig. 10A–E

Xyleborus percuneolus Schedl, 1951: 85.
Xyleborinus percuneolus (Schedl): Wood and Bright 1992: 809.
Cryptoxyleborus percuneolus (Schedl): Beaver and Hulcr 2008: 145.

##### Diagnosis.

Minute, the smallest *Cryptoxyleborus* species, 1.4 mm long; elongate, 2.55 times longer than wide; body dull, glabrous except for mycangial tuft along elytral base, red brown to brown in color; antennal club approximately circular, first segment smaller than second (type 4; Hulcr et al. 2007), pronotum from dorsal view elongated basic shape (type 7; Hulcr et al. 2007), anterior margin round, armed with minute serrations, pronotal disc alutaceous, shagreened, from lateral view elongate, with low summit (type 7; Hulcr et al. 2007); elytra stout, 1.75 times longer than pronotum, base sinuate without mycangial pits, disc shining, interstriae distinctly seriate punctate, declivital striae and interstriae granulate, never bearing hooked tubercles, elytra gradually tapering from midpoint to apex.

**Figure 10.**
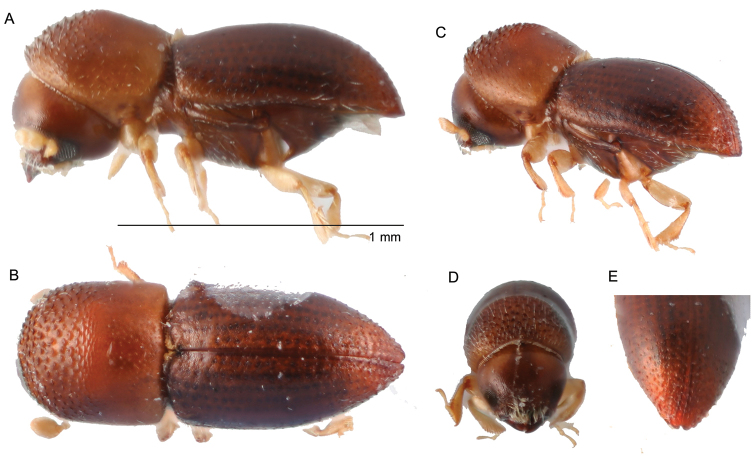
*Cryptoxyleboruspercuneolus* (Schedl) **A** lateral view **B** dorsal view **C** posterolateral view **D** front **E** declivity.

##### Material examined.

THAILAND, Ton Nga Chang Wildlife Sanctuary, Songkhla Province, 6°59'32.1"N, 100°08'57.8"E, tropical rainforest, ethanol-baited trap, 01.vi.2015 (1) (W. Sittichaya).

##### Distribution.

Indonesia (Java), Malaysia (Sabah). New to Thailand.

##### Biology.

Like other species of *Cryptoxyleborus*, its hosts are probably confined to trees of the family Dipterocarpaceae (Beaver and Hulcr 2008). However, no host trees have yet been recorded. One gallery system investigated in an undetermined tree comprised an unbranched entrance tunnel leading to a single terminal brood chamber enlarged in the longitudinal plane, with multiple tunnels extending further into the wood (Beaver and Hulcr 2008).

##### Remark.

This species can be distinguished from all other *Cryptoxyleborus* recorded in Thailand by its minute size (1.4 mm), elytral base sinuate and lacking mycangial pits.

### *Cyclorhipidion* Hagedorn, 1912: 355

#### Cyclorhipidion vigilans

Taxon classificationAnimaliaColeopteraCurculionidae

(Schedl, 1939)

Fig. 11A–E

Xyleborus vigilans Schedl, 1939: 43.
Cyclorhipidion vigilans (Schedl): Wood and Bright 1992: 704.

##### Diagnosis.

Large, 5.5 mm long (*N* = 5); elongate, 2.45−2.48 times longer than wide; pronotum with less vestiture than elytra, elytra densely covered with interstrial setae, density of vestiture varies; brown to dark brown in color; pronotum from dorsal view basic shape, anterior margin subquadrate, sides parallel (type 3; Hulcr et al. 2007), 1.55 times longer than wide, from lateral view lateral view rounded, robust (type 5; Hulcr et al. 2007), anterior margin extended anteriad, armed with 4−6 medium sized serrations, anterior half of pronotum asperate, base shagreened; elytra elongate, gradually tapering from base posteriorly, discal striae punctate, clearly impressed, interstriae two times broader than striae, covered with fine hair-like setae, setae longer posteriorly, discal striae and interstriae 1−3 laterally diverging from suture at basal third, declivital striae irregularly punctate, interstria granulate, posterolateral carina granulate, extending from apex to interstriae 7.

**Figure 11.**
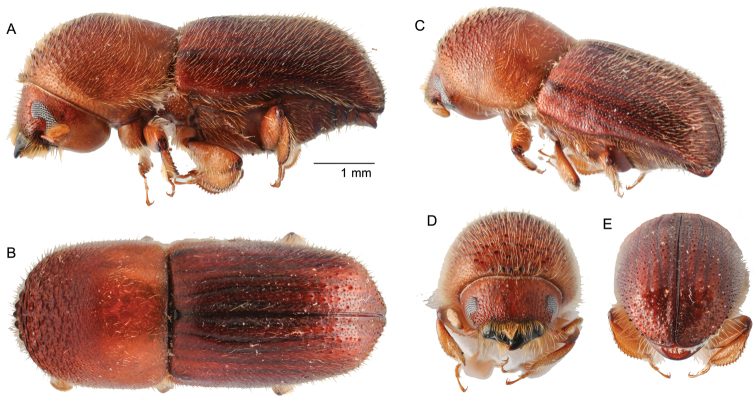
*Cyclorhipidionvigilans* (Schedl, 1939) **A** lateral view **B** dorsal view **C** posterolateral view **D** front **E** declivity.

##### Material examined.

THAILAND, Ton Nga Chang Wildlife Sanctuary, Songkhla Province, 6°59'32.1"N, 100°08'57.8"E, tropical rainforest, ethanol-baited trap, 01.i.2014 (1), 01.iii.2014 (1), 01.iv.2015 (3) (W. Sittichaya).

##### Distribution.

East and West Malaysia, Indonesia (Java). New to Thailand.

##### Biology.

Recorded only from ‘kalapa tjoeng’ (*Horsfieldiaglabra* (Reinw. ex Blume) Warb.) (Myristicaceae) (Schedl 1939).

##### Remarks.

This species can be distinguished by the large size, and anterior margin of pronotum extended anteriad and armed with 4−6 medium sized serrations; elytra elongate tapering from base to angularly rounded apex, discal striae 1‒3 impressed, interstriae 2 widened and outwardly curved in middle of disc, interstriae 3 correspondingly narrowed, interstriae granulate on upper part of declivity. This species is similar to species in the genus *Fortiborus*, but the body is densely covered with long hairs and the lower part of the eye is larger than the upper part.

## Discussion

The xyleborine fauna of Thailand is the most well-known and diverse in South-East Asia with 146 species previously recorded. The ten additional records presented here illustrate both the richness of this fauna and how much remains to be discovered, particularly in the south of the country. Three of the species we reported were already known from South-East Asia, while the remaining seven are shared with the Indo-Malayan fauna. We have not included a key to the xyleborines of Thailand in this paper because a monograph of the tribe in East and South-East Asia is currently being prepared (Smith, Beaver, Cognato, in prep.). This will include a key to the xyleborines of the whole of this region. Most xyleborine species have broad distributions, and this large-scale monograph will provide the necessary information and tools to identify genera and species, and assist in the recognition of new taxa. This publication reports new records of Xyleborini found during an intensive survey of Thai forests. Additional new records of species from other tribes will be covered in future papers as further study is necessary.

## Supplementary Material

XML Treatment for Amasa beesoni

XML Treatment for Amasa cylindrotomica

XML Treatment for Amasa opalescens

XML Treatment for Arixyleborus hirsutulus

XML Treatment for Beaverium latus

XML Treatment for Cnestus protensus

XML Treatment for Coptodryas quadricostata

XML Treatment for Cryptoxyleborus confusus

XML Treatment for Cryptoxyleborus percuneolus

XML Treatment for Cyclorhipidion vigilans
